# Effect of parathyroid hormone on the structural, densitometric and failure behaviors of mouse tibia in the spatiotemporal space

**DOI:** 10.1371/journal.pone.0219575

**Published:** 2019-07-10

**Authors:** Yongtao Lu, Jintao He, Hanxing Zhu, Yongxuan Wang

**Affiliations:** 1 Department of Engineering Mechanics, Dalian University of Technology, Dalian, China; 2 State Key Laboratory of Structural Analysis for Industrial Equipment, Dalian University of Technology, Dalian, China; 3 School of Engineering, Cardiff University, Cardiff, United Kingdom; 4 Affiliated Zhongshan Hospital of Dalian University, Dalian, Liaoning, China; Nanjing Medical University, CHINA

## Abstract

Parathyroid hormone (PTH) is an anabolic bone drug approved by the US Food and Drug Administration (FDA) to treat osteoporosis. However, previous studies using cross-sectional designs have reported variable and sometimes contradictory results. The aim of the present study was to quantify the localized effect of PTH on the structural and densitometric behaviors of mouse tibia and their links with the global mechanical behavior of bone using a novel spatiotemporal image analysis approach and a finite element analysis technique. Twelve female C57BL/6J mice were divided into two groups: the control and PTH treated groups. The entire right tibiae were imaged using an *in vivo* micro-computed tomography (μCT) system eight consecutive times. Next, the *in vivo* longitudinal tibial μCT images were rigidly registered and divided into 10 compartments across the entire tibial space. The bone volume (BV), bone mineral content (BMC), bone tissue mineral density (TMD), and tibial endosteal and periosteal areas (TEA and TPA) were quantified in each compartment. Additionally, finite element models of all the tibiae were generated to analyze the failure behavior of the tibia. It was found that both the BMC and BV started to increase in the proximal tibial region, and then the increases extended to the entire tibial region after two weeks of treatment (*p* < 0.05). PTH intervention significantly reduced the TEA in most tibial compartments after two weeks of treatment, and the TPA increased in most tibial regions after four weeks of treatment (*p* < 0.05). Tibial failure loads significantly increased after three weeks of PTH treatment (*p* < 0.01). The present study provided the first evidence of the localized effect of PTH on bone structural and densitometric properties, as well as their links with the global mechanical behaviors of bone, which are important pieces of information for unveiling the mechanism of PTH intervention.

## Introduction

Osteoporosis is a major bone disease that increases the risk of bone fracture and consequently largely affects the aging members of society [**1**]. Parathyroid hormone (PTH) is an anabolic bone drug approved by the U.S. Food and Drug Administration (FDA) to treat osteoporosis. However, variable and sometimes contradictory results have been reported in previous studies regarding the effects of PTH on bone [**2–4**]; the reason for these results may be that the mechanism of PTH on bone, especially in the entire spatiotemporal space of bone, is still not fully understood.

A well-developed preclinical study using female mice has been established as an approach to investigate the effects of PTH on bone properties. However, most previous studies have used a cross-sectional study design [**5–7**], in which different groups of mice were killed at different time points, and then the results from different mice were averaged and statistically analyzed. Two main issues are associated with these cross-sectional studies. First, due to the intersubject variances, a large number of animals are usually needed to remove the influence of intersubject variances, which might be the main reason for the controversial results reported in the literature. Second, cross-sectional studies prevent longitudinal monitoring of bone changes in the spatiotemporal space, which is crucial information for providing an in-depth understanding of the PTH intervention.

The technique of *in vivo* micro-computed tomography (μCT) imaging allows for noninvasive and longitudinal monitoring of changes in the same bone and consequently eliminates the influence of intersubject variances [**8–13**]. Using this technique, a number of studies have investigated the effects of interventions on bone properties [**9–13**]. However, only a small region of bone has been used in these studies, such as the proximal tibia [**9–11**] or the tibial midshaft [**12–13**]. The authors’ previous studies have showed that localized changes in bone properties may not be sufficient to lead to changes in the mechanical properties of bone (i.e., stiffness and failure load) [**14–15**]. Therefore, a region of bone may not be representative of the behavior of the entire bone. To address this issue, researchers from Switzerland investigated longitudinal changes of bone properties over the entire caudal vertebrae [**16–17**]. However, an investigation using long bones, e.g., the tibia, is still missing. An analysis on long bones is necessary for two main reasons. First, the long bones (tibia and femur) are the main sites supporting weight, which puts them at a higher fracture risk during daily activities. Second, the effect of PTH on long bones may be different from that on other sites, such as the caudal vertebrae.

Previously, we have developed a novel spatiotemporal imaging analysis approach, which can quantify the changes of bone properties in the spatiotemporal space with a high accuracy [**18**]. The approach has been used to analyze the longitudinal effects of PTH on the morphological, densitometric and mechanical properties of bone [**15**]. However, many bone adaptation mechanisms still need to be explored further using this novel analysis approach. For example, an analysis of bone structural parameters, especially the tibial periosteal and endosteal areas (TPA and TEA), is still missing. The quantification of TPA and TEA in the spatiotemporal space can unveil important mechanisms about how bone adapts itself in terms of bone structure.

On the other hand, based on the *in vivo* longitudinal μCT images, finite element (FE) models can be generated to noninvasively analyze the mechanical properties of bone. The FE method has been widely used in bone research [**12–16**], and previous studies have showed that FE models can predict the mechanical properties of bone well, including the stiffness and failure load [**19, 20**]. However, to the authors’ knowledge no previous study has used the FE method to investigate the longitudinal effects of PTH on bone failure properties. Understanding the effect of PTH on bone failure behavior and its link with localized changes in the structural and densitometric parameters of bone could help improve the ability of bones to resist fractures, which is the ultimate goal of PTH treatment.

The aim of this study was to investigate the longitudinal effects of PTH intervention on localized structural and densitometric properties of bone and their links with the global mechanical properties of bone using *in vivo* μCT imaging, spatiotemporal imaging analysis and the finite element analysis technique.

## Material and methods

### *In vivo* μCT imaging

Twelve 13-week-old female C57BL/6 mice were purchased and housed in the affiliated Zhongshan Hospital of Dalian University with a twelve-hour light/dark cycle at 22°C and free access to food and water. The mice were divided into control and PTH treated groups, with six mice in each group. At 18 weeks of age the mice started to receive a daily injection of either PTH or a vehicle. In the control group (Wild), the mice (N = 6) received the injection of the vehicle in order to remove the influence of surgery on the results, while in the PTH treated group (WildPTH), the mice (N = 6) received a daily injection of PTH (hPTH 1–34, Bachem, Bubendorf, Switzerland) at 100 ng/g/day, seven days a week. All the injections were given until week 22 (the end of experiment). The entire right tibiae of the mice were imaged using the *in vivo* μCT system at ages of 14, 16, 17, 18, 19, 20, 21 and 22 weeks with an isotropic image voxel size of 10.4 μm, a voltage of 55 keV, a tube current of 145 μA and an integration time of 200 ms.

### Image processing and the spatiotemporal analysis approach

**Fig 1:**
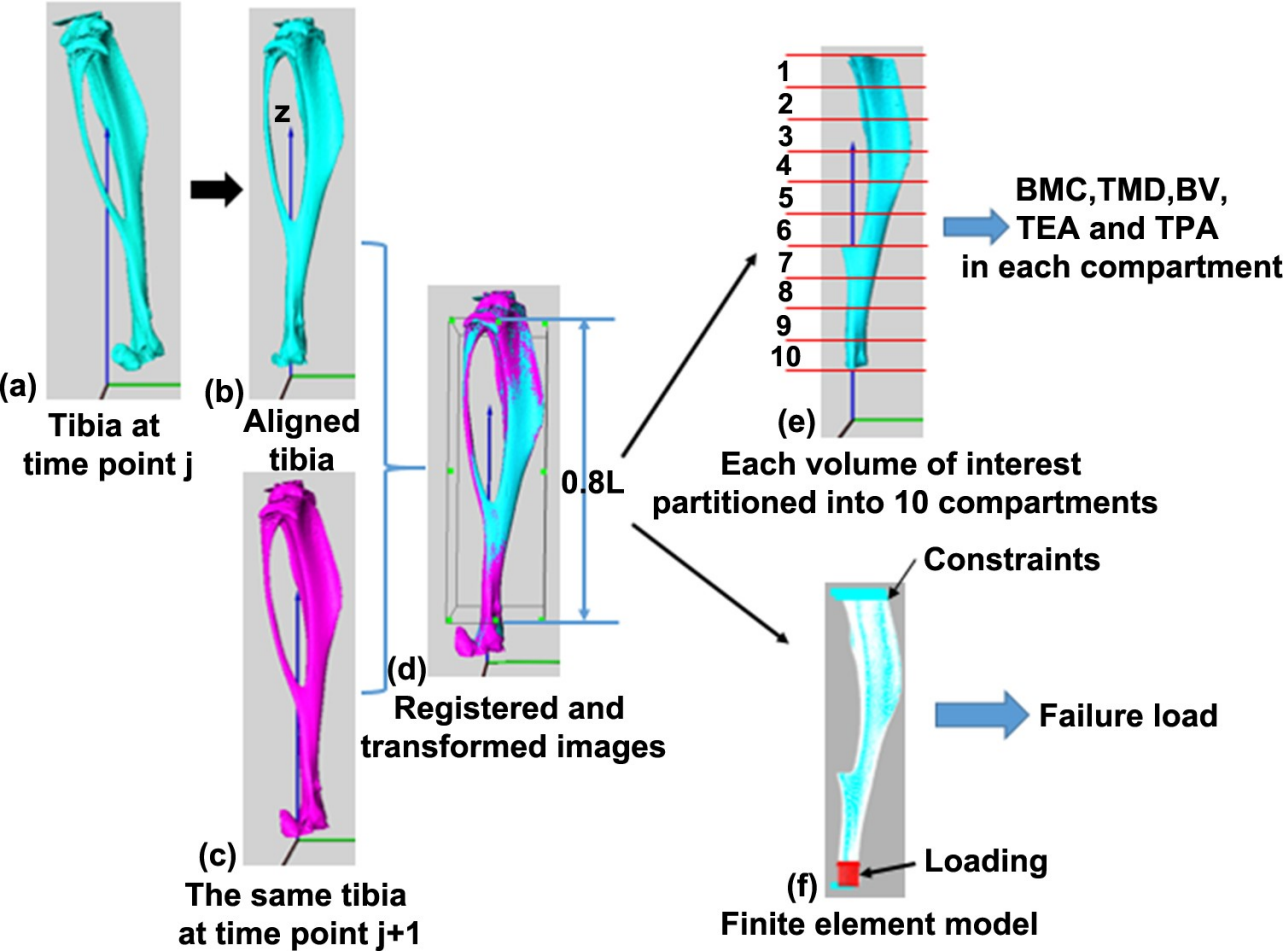
Schematic representation of the calculation of the structural, densitometric and mechanical properties of the tibia. (a-d) Rigid registration of the mouse tibiae using a stepwise approach; (e) quantification of the structural (BV, TEA and TPA) and densitometric (BMC and TMD) properties of the tibia; (f) calculation of the mechanical (failure load) properties of the tibia.

The *in vivo* μCT image datasets were processed using the previously developed spatiotemporal quantification method [**14**, **15**, **18**]. In brief, a rigid registration was used to align all the tibiae into the same orientation using the image processing software—Amira (v5.4.3, FEI Visualization Sciences Group, France). First, one tibia chosen from the baseline scans (week 14) was taken as the reference, and the proximal-distal axis of this tibia was aligned along the z-axis (**Fig 1B**). Then, the follow-up scans of the same tibia were rigidly registered to the reference tibia in a stepwise manner using a Quasi-Newton optimizer and Euclidean distance as the similarity measure (Amira 5.4.3) **[18]**. For example, a tibia scanned at time point *j*+1 was rigidly registered to the same tibia scanned at the previous time point, i.e., time point j (**Fig 1B–1D**). The stepwise approach is utilized to minimize the influence of tibial growth on the results **[15]**. For the tibiae from mice other than the reference mouse, the baseline (week 14) datasets were first rigidly registered to the reference tibia, and then the follow-up scans of these tibiae were rigidly registered to their corresponding baseline tibiae in a stepwise manner. Following the rigid registrations, the image datasets were transformed to the new positions and resampled using Lanczo’s kernel **[18]**. In each resampled image dataset the tibial length (L) was measured as the distance from the most proximal tibial voxel to its most distal voxel. To automate the entire processing procedure, a region of 80% of the tibia starting from the distal end of the proximal growth plate was cropped out and selected as the volume of interest (VOI) (**Fig 1**) [**15**]. To quantify bone adaptations in the spatial scale, the VOI were partitioned into ten compartments, each with an equal length in the z-direction, using in-house developed MATLAB code (v2015a, The MathWorks, Inc. Natrick, MA) (**Fig 1E**) [**14**].

The following bone parameters were quantified in each compartment using standard procedures: bone mineral content (BMC, units of mg hydroxyapatite (HA)), tissue mineral density (TMD, units of mg HA/cm^3^), bone volume (BV, units of mm^3^), tibial endosteal area (TEA, units of mm^2^) and tibial periosteal area (TPA, units of mm^2^) (**Fig 1E**) [**14–15**,**18**,**21–22**]. In brief, the grayscale images were first smoothed using a Gaussian filter (convolution kernel = [3 3 3], standard deviation = 0.65) to reduce the influence of image noise. Then, the HA-equivalent BMD value in each image voxel was calculated from the CT grayscale values using the calibration function provided by the manufacturer. The μCT scanner was checked weekly using a five-rod densitometric calibration phantom. The grayscale images were binarized using a threshold value of 25.5% of the maximal grayscale value of the image [**23**]. Based on the binary images, bone masks, which are regions occupied by bone, were identified. The BMC values in each compartment were calculated as the total bone mineral content over the masked bone region, and the total tibial BMC was calculated as the sum of all BMC values in the 10 compartments. The TMD was calculated as the mean mineral density within the masked bone regions. BV was calculated as the total bone volume over the masked bone regions [**14,15**]. Regarding the calculation of TEA and TPA, a well-established automatic image segmentation approach was first used to segment the endosteal and periosteal surfaces of the mouse tibia (MATLAB 2015a, The MathWorks, Inc. Natrick, MA) [**24**]. Next, the TEA and TPA were calculated as the areas enclosed by the endosteal and periosteal surfaces of the mouse tibia, respectively [**14,21**].

In the results section of this study the data (BMD, TMD, BV, TEA and TPA) are visualized and presented in two different ways. First, to visualize the longitudinal changes in the tibial TEA and TPA for both the Wild and WildPTH groups, the TEA and TPA values at week *j* (*j* = 16,…, 22) in each compartment are presented as the difference between the values in week 14 and week j, normalized with respect to the baseline (week 14) values in the Wild and WildPTH groups, respectively. Second, to visualize the effect of PTH on the various bone parameters (BMC, TMD, BV, TEA and TPA), the changes in bone parameters (BP) in each compartment are represented by their mean relative percentage difference (**δD**%_***j***_) between the treatment (WildPTH) and control (Wild) groups [**14, 15, 22**]:

δD%j=(ΔBj−ΔAj)REFj×100 (1)

where,

$${\Delta A}_{j}=\frac{\sum_{i=1}^{n1}\left({BP}_{i,j}^{Wild}-{BP}_{i,14}^{Wild}\right)}{n1}$$

$${\Delta B}_{j}=\frac{\sum_{i=1}^{n2}\left({BP}_{i,j}^{WildPTH}-{BP}_{i,14}^{WildPTH}\right)}{n2}$$

$${REF}_{j}=\frac{\sum_{i=1}^{n1}{BP}_{i,j}^{Wild}}{n1}$$

*n*1 and *n*2 are the numbers of mice in the Wild and WildPTH groups, j represents the week index (j = 16, …, 22), *i* represents the index number of each mouse and ***BP*** represents the bone parameters of BMC, TMD, BV, TEA and TPA.

### Reproducibility of the measurements of bone parameters

Experimental measurements are always associated with measurement errors. To detect any significant effects of an intervention, the measurement errors need to be quantified and removed when interpreting the data. In the present study, the measurements errors associated with the BV, TEA, TPA, BMC and TMD were quantified. For this aim, eight tibiae from 14-week-old C57Bl/6 female mice were used and scanned four times each in the *in vivo* μCT scanner using the same setup as used for the longitudinal scanning, i.e., 10.4 μm, 55 keV, 145 μA and 200 ms. In the reproducibility study the mouse tibiae were repositioned in the sample holder between scans in order to simulate the longitudinal study design. The repeated image datasets were processed using the procedures developed in the present study for processing the longitudinal images (**Section 2.2**). The same bone measurements, i.e., BV, TEA, TPA, BMC and TMD, were quantified from the processed repeated images. Precision errors (PEs) and intraclass correlation coefficients (ICCs) were then calculated to characterize the reproducibility of these bone measurements [**18**, **22**]. The precision errors (PEs) were expressed as the coefficients of variations (CV) (PE_%CV_) [**25**]:

$${PE}_{\%cv}=\sqrt{\sum_{j=1}^{m}{\%CV}_{j}^{2}/m}$$ (2)

with

$$\%{CV}_{j}=\frac{{SD}_{j}}{{\bar{x}}_{j}}\times 100\%$$ (3)

where m is the subject number (m = 8 in the current study) and ${\bar{x}}_{j}$ is the mean of all *x*_*ij*_ for subject j.

To determine the accuracy of the PEs, confidence intervals (CIs) were determined for each of the PE_%CV_ values using a chi-squared distribution (*χ*^2^) [**26**].

$$\frac{df}{\chi_{1-\frac{\alpha }{2},df}^{2}}{PE}_{{\%CV}^{2}}<\sigma^{2}<\frac{df}{\chi_{\frac{\alpha }{2},df}^{2}}{PE}_{{\%CV}^{2}}$$ (4)

where df is the total degrees of freedom (df = 24 in the current study).

The ICC was expressed as the ratio of the intersubject variance over the population variance [**8**]:

$$ICC=\frac{F_{0}-1}{F_{0}+(n-1)}$$ (5)

where F_0_ is the ratio of the mean squares between subjects over the residual mean squares within subjects, and n is the number of repetitions (n = 4 in this study). The ICC values vary between 0 and 1, where 1 denotes perfect reproducibility.

### Finite element analysis

Following the procedure established previously [**14**,**15**], finite element models of mouse tibiae were generated to analyze the effects of PTH intervention on the mechanical behavior of mouse tibia (failure load). Linear elastic, homogeneous FE models were created by converting each bone voxel into an 8-node hexahedron element after removing all the unconnected bone islands in the processed binary images of the tibial VOI [**14**]. Young’s modulus of 14.8 GPa and Poisson’s ratio of 0.3 were defined, and a loading scenario of uniaxial compression was simulated, i.e., all the degrees of freedom of the nodes in the four most proximal layers of the tibial VOI were fixed and a displacement of 1.00 mm was applied to the nodes in the four most distal layers (**Fig 1F**)[**14**,**15**]. Because the bone failure strength is of high interest to both surgeons and patients and is highly linked to bone fracture risk [**27**], the tibial failure loads were investigated and calculated using the maximum principal strain criterion. The failure load was calculated as the value when 5% of bone tissues in the region of investigation exceeded the principal strain limits, which are 7300 με for tensile strain and 10,300 με for compressive strain [**28**]. According to Saint-Venant’s principle, the results in regions close to the boundary conditions are influenced by the boundary conditions. Therefore, to remove the influence of boundary conditions on the calculated failure load, some regions in the two ends of the mouse tibia were removed, and the rest were taken as the region of investigation [**14**,**15**]. The FE models were solved using ANSYS (V15.0, ANSYS, Inc., Cannonsburg, P.A., USA) on a workstation with the following specifications: Intel Xeon E5-2630 v3@2.40 GHz, 512 GB RAM.

### Statistical analysis

Using the mean and standard deviation (SD) data of the Wild and WildPTH groups, the effect of PTH treatment on the structural parameters (BV, TEA and TPA), densitometric parameters (BMC and TMD) and the FE predicted failure loads of bone were analyzed using the analysis of covariance (ANCOVA) by taking the corresponding baseline values at week 14 as the covariables. The ANCOVA was performed using the statistical analysis tool—R software (https://www.r-prject.org/). The probability of type I error was set to α = 0.05, which means *p* < 0.05 was considered statistically significant. To visualize the adaptation of the TEA and TPA and the effects of PTH on the bone parameters in the spatiotemporal space, the normalized differences between groups, the calculation process of which was described in the third paragraph of Section 2.2 in the present paper, are presented, and the statistical significance is marked in the corresponding locations. Linear regression equations and the coefficients of determination (R^2^) were computed for the relationships between the tibial BMCs and the FE predicted failure loads.

## Results

### The measurement errors associated with the image processing pipeline

The precision errors (PEs) and the intraclass correlation coefficients (ICCs) for the bone measurements are presented in **Table 1**. The mean PEs ranged from 1.23% to 1.98% for BV, from 1.11% to 1.41% for TMD, from 1.25% to 2.01% for BMC, from 0.67 to 1.28% for TEA, and from 0.46% to 0.88% for TPA (**Table 1**). For each bone parameter, the PEs are similar across the tibial length (**Table 1)**, which shows a homogenous effect of the reproducible measurements. Taking into account the 95% confident intervals of PEs, the precision errors for the regional BV, TMD, BMC, TEA and TPA are chosen to be 2.5%, 2.0%, 2.5%, 1.5% and 1.0%, respectively, for the subsequent analysis in the present study. Therefore, only differences smaller than or larger than these values can be interpreted as between-group differences. Regarding the ICCs, they are large for the regional BMC, BV, TEA and TPA (0.86 to 0.99) (**Table 1**), which means that the intersubject differences are larger than the repeated-scan differences for these measurements. The ICCs for the regional TMD are small (0.26 to 0.84) (**Table 1**), which could be because the differences between mice are small for the regional TMD.

**Table 1:** Reproducibility of BV, TMD, BMC, TEA and TPA expressed in mean precision errors (PE) as coefficients of variation (the 95% confident intervals shown in Parentheses), and the intraclass correlation coefficients (ICC) (C01 –C10 corresponds to the proximal to distal sides of the tibia, see Fig 1E).

	BV		TMD		BMC		TEA		TPA	
	PE [%]	ICC	PE [%]	ICC	PE [%]	ICC	PE [%]	ICC	PE [%]	ICC
C01	1.98	0.94	1.41	0.84	2.01	0.96	0.93	0.97	0.73	0.98
	(1.6 2.6)		(1.1 1.9)		(1.7 2.5)		(0.8 1.2)		(0.6 0.9)	
C02	1.66	0.95	1.34	0.70	1.64	0.96	0.87	0.97	0.60	0.99
	(1.3 2.2)		(1.1 1.8)		(1.4 2.3)		(0.7 1.1)		(0.5 0.8)	
C03	1.69	0.94	1.43	0.54	1.53	0.95	1.03	0.97	0.67	0.98
	(1.4 2.2)		(1.2 1.9)		(1.3 2.2)		(0.9 1.4)		(0.5 0.9)	
C04	1.53	0.90	1.35	0.64	1.45	0.94	1.28	0.96	0.61	0.97
	(1.2 2.0)		(1.1 1.8)		(1.2 2.0)		(1.0 1.6)		(0.5 0.8)	
C05	1.29	0.94	1.17	0.56	1.47	0.94	0.67	0.99	0.58	0.98
	(1.1 1.7)		(1.0 1.5)		(1.2 2.0)		(0.5 1.0)		(0.5 0.8)	
C06	1.20	0.93	1.05	0.50	1.35	0.94	0.72	0.99	0.52	0.97
	(1.0 1.6)		(0.9 1.4)		(1.1 1.9)		(0.6 1.0)		(0.4 0.7)	
C07	1.23	0.92	1.15	0.36	1.25	0.93	0.87	0.99	0.54	0.98
	(1.0 1.6)		(0.9 1.5)		(1.0 1.8)		(0.7 1.1)		(0.4 0.7)	
C08	1.19	0.95	1.35	0.26	1.95	0.90	1.01	0.99	0.73	0.94
	(1.0 1.6)		(1.1 1.8)		(1.6 2.4)		(0.8 1.2)		(0.6 1.0)	
C09	1.24	0.94	1.52	0.26	1.72	0.92	1.07	0.99	0.46	0.98
	(1.0 1.6)		(1.2 2.0)		(1.4 2.1)		(0.9 1.3)		(0.4 0.6)	
C10	1.94	0.86	1.11	0.52	1.91	0.90	1.19	0.99	0.88	0.97
	(1.6 2.6)		(0.9 1.5)		(1.6 2.5)		(0.9 1.4)		(0.7 1.2)	

### Effect of PTH on the tibial BV, TMD, BMC, TEA and TPA

**Fig 2:**
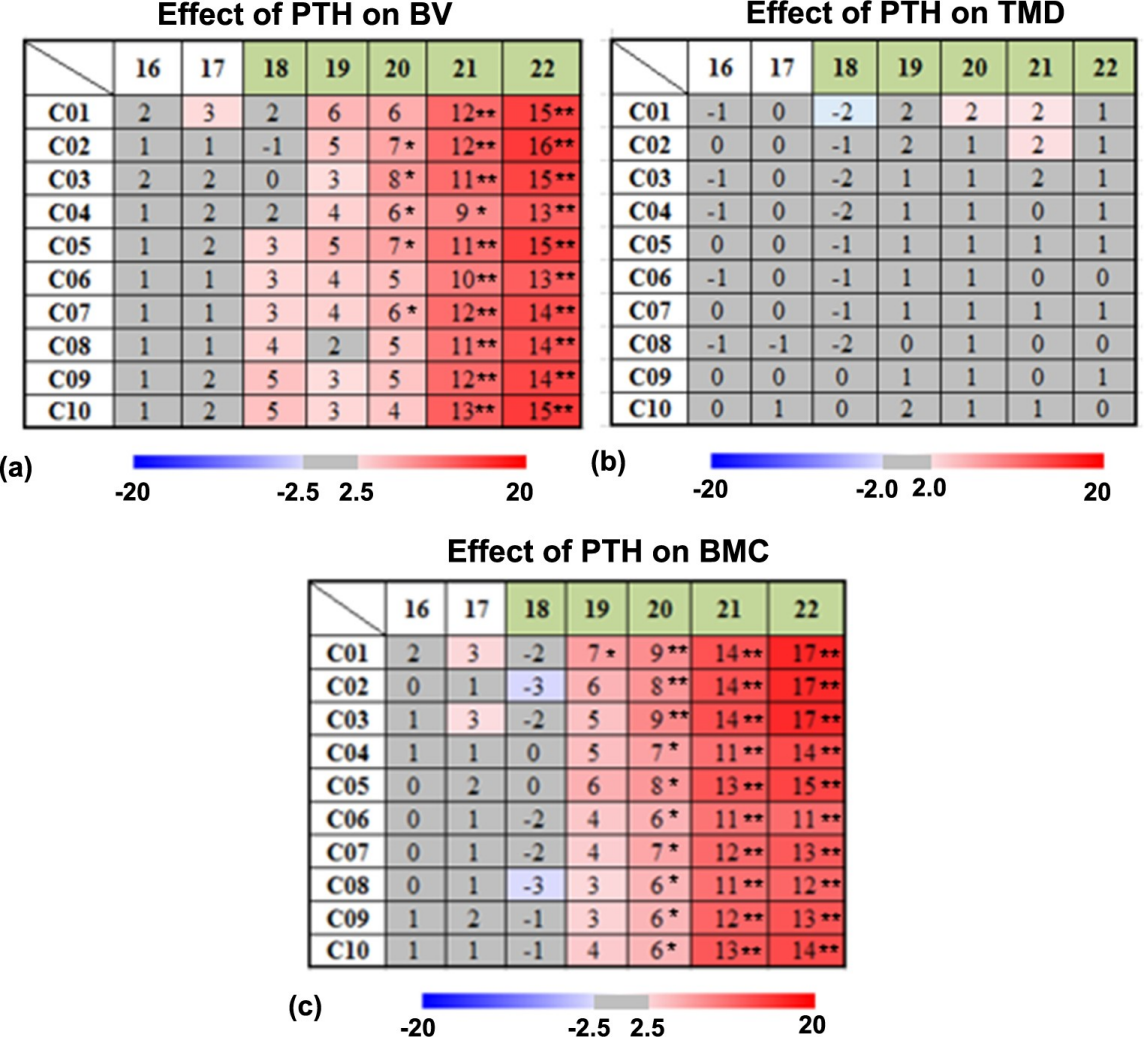
Longitudinal effects of PTH treatment on tibial BV, TMD and BMC in the spatiotemporal space. The data are presented as the mean relative percentage difference (δD%_*j*_) (Eq 1) between the Wild and WildPTH groups for the 10 compartments along the proximal-distal tibial axis (* *p* < 0.05, ** *p* < 0.01) (intermittent PTH treatment started in week 18 and lasted until week 22, which is labeled in green in the Figure).

The spatiotemporal analysis showed significant effects of the PTH treatment on the tibial BV in some tibial compartments (C02, C03, C04, C05 and C07) after two weeks of PTH intervention, e.g., at week 20 in compartment C02, the BV in the WildPTH group increased significantly by 7% with respect to the value in the Wild group (*p* <0.05) (**Fig 2A**). After three weeks of treatment (week 21), the effect extended to all the tibial compartments, and the magnitude of the BV increase ranged from +9% to +13% in the 10 compartments (all *p* < 0.05) (**Fig 2A**). The spatiotemporal analysis showed there was no effect from PTH intervention on the regional tibial TMD (all *p* > 0.05) (**Fig 2B**). The effect of PTH intervention on the regional tibial BMC was similar to that on BV (**Fig 2C**), but the effect of PTH on the BMC started one week earlier, i.e., significant effects from PTH on the tibial BMC were found after one week of PTH treatment. For example, at week 19 in compartment C01, BMC in the WildPTH group increased significantly by 7% with respect to the value in the Wild group (*p* <0.05) (**Fig 2C**). After two weeks of treatment (week 20), the effect of PTH on the BMC extended to all the tibial compartments, and the magnitude of the BMC increase ranged from +6% to +9% in the 10 compartments (all *p* < 0.05) (**Fig 2C**).

**Fig 3:**
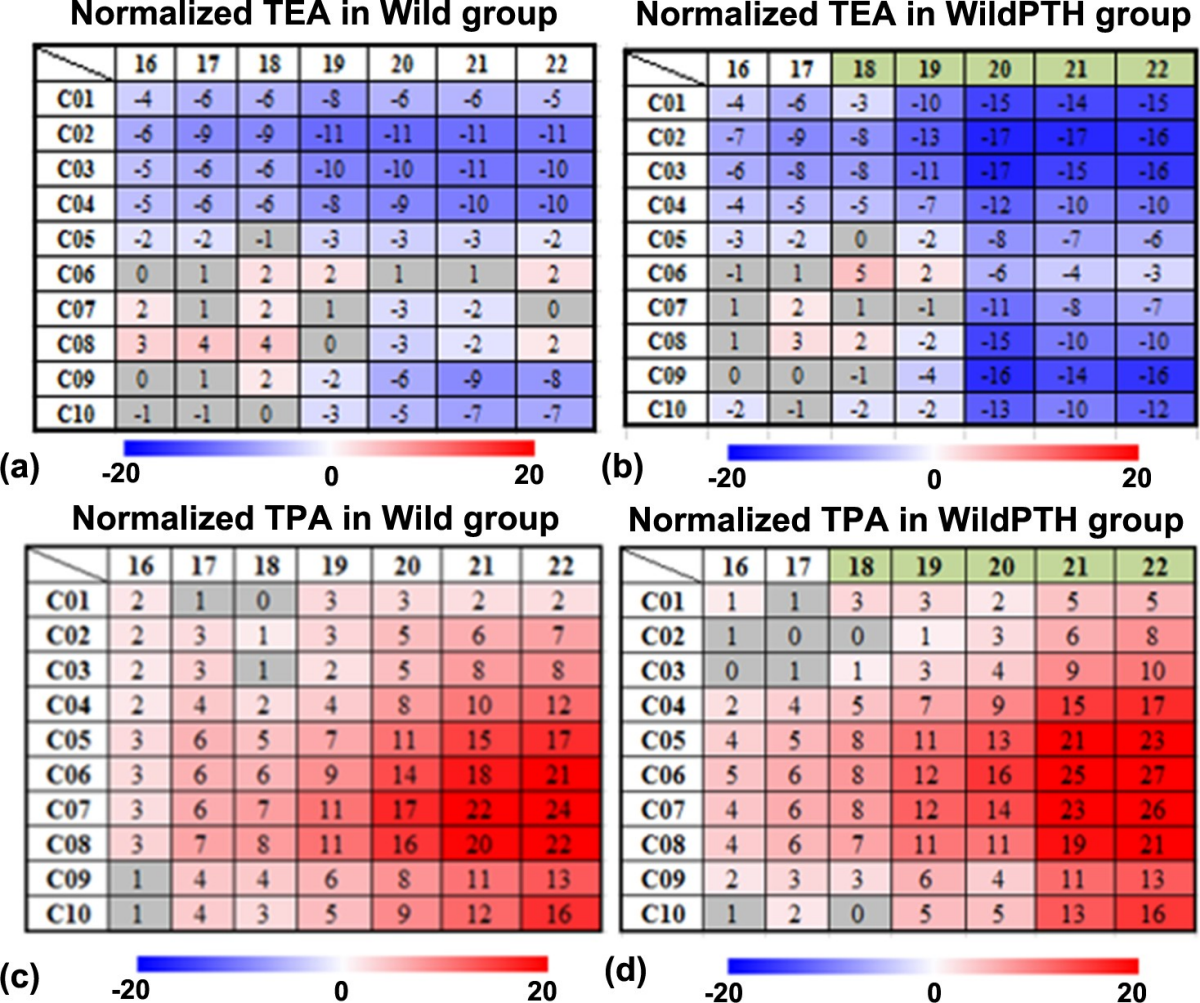
The longitudinal adaptation of the endosteal and periosteal areas (TEA and TPA) of mouse tibiae in the spatiotemporal space in both the Wild and WildPTH groups. The data (units in percentages) are presented as the mean changes normalized with respect to the baseline values at week 14 (intermittent PTH treatment started in week 18 and lasted until week 22, which is labeled in green in the Figure).

**Fig 4:**
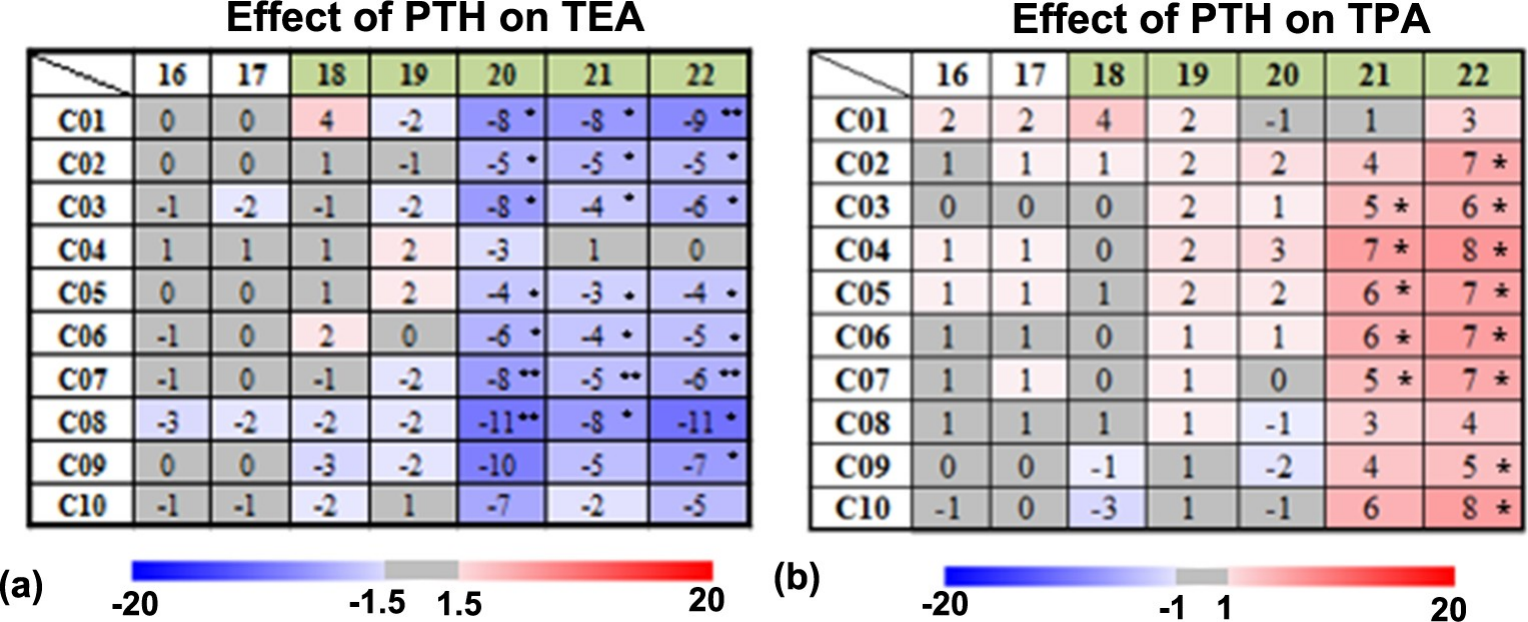
Longitudinal effects of PTH treatment on the tibial TEA and TPA in the spatiotemporal space. The data are presented as the mean relative percentage difference (δD%_*j*_) between the Wild and WildPTH groups (* *p* < 0.05, ** *p* < 0.01).

From week 14 to week 22, the TEA decreased in the Wild group in some tibial regional compartments, especially in the proximal compartments (compartments C01 to C05) (**Fig 3A**). The rate of TEA decrease increased in the proximal tibial compartments (C01 to C03) after one week of PTH intervention (week 19), e.g., at week 19 in C01, with respect to the values at week 14, the TEA decreased by 8% in the Wild group, and the decrease rate increased to 10% in the WildPTH group (**Fig 3A and 3B**). The increasing TEA decrease rate extended to all the compartments after two weeks of PTH treatment (**Fig 3A and 3B**). PTH intervention significantly reduced the TEA in most tibial compartments after two weeks of treatment (week 20), e.g., at week 20 in compartment C01, the TEA in the WildPTH group decreased significantly by 8% with respect to the value in the Wild group (*p* <0.05) (**Fig 4A**).

From week 14 to week 22, the TPA increased in the Wild group in some tibial regional compartments, especially in the midshaft region (C05 to C08 compartments) (**Fig 3C**). The rate of TPA increase increased in the midshaft region (C04 to C07) after one week of PTH intervention (week 19), e.g., at week 19 in C04, with respect to the values at week 14, the TPA increased by 4% in the Wild group, and the increase rate increased to 7% in the WildPTH group (**Fig 3C and 3D**). The increasing TPA increase rate extended to most tibial compartments after three weeks of PTH treatment (**Fig 3C and 3D**). PTH intervention significantly increased the TPA in most tibial compartments after four weeks of treatment (week 22), e.g., at week 22 in compartment C03, the TPA in the WildPTH group increased significantly by 6% with respect to the value in the Wild group (*p* <0.05) (**Fig 4B**).

### Tibial failure load, total BMC and their correlations

**Fig 5:**
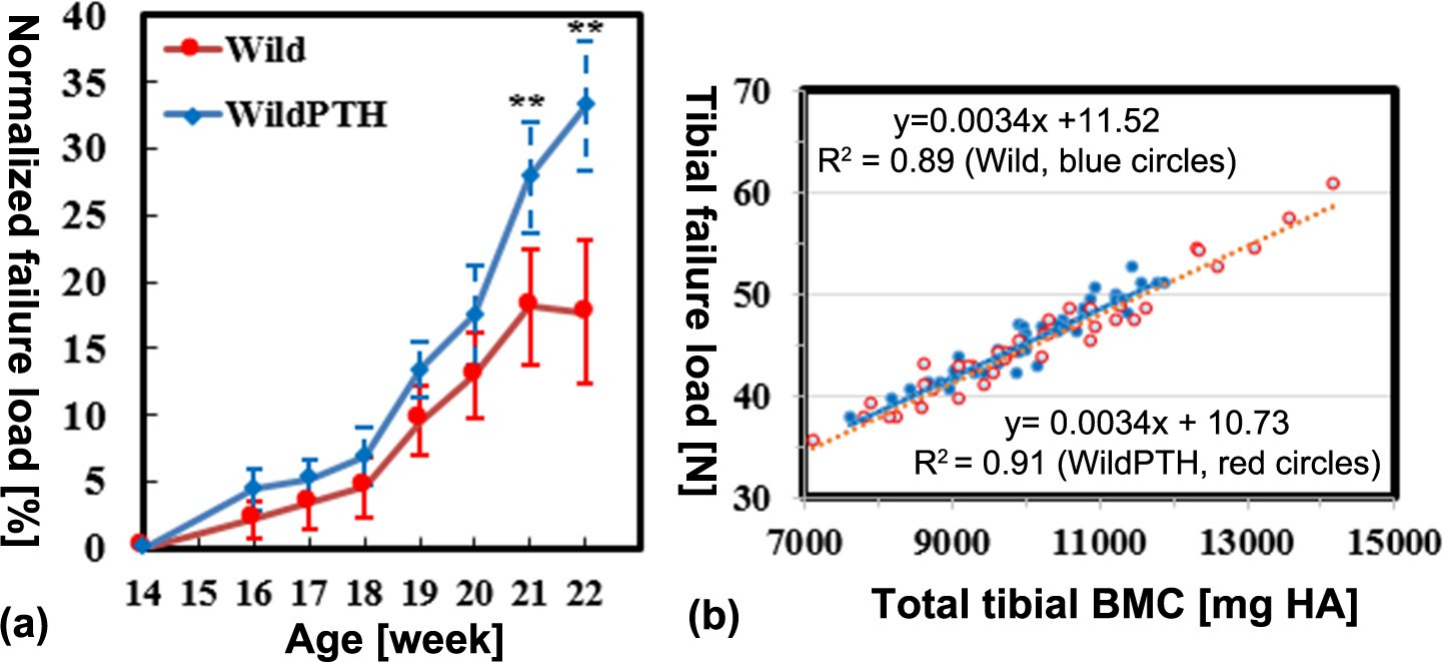
(a) Longitudinal effects of PTH intervention on the FE predicted tibial failure load. The data are presented as the mean standard deviation of the normalized data with respect to baseline (* *p* < 0.05, ** *p* < 0.01). (b) Linear regression analysis for the relationships between the FE predicted failure loads and tibial BMCs (Wild group in blue circles and WildPTH group in red circles).

The normalized tibial failure loads predicted from the FE analysis increased from week 14 until week 22 in both the Wild and WildPTH groups (**Fig 5A**). The failure loads in the WildPTH group were significantly higher than those in the Wild group after three weeks (week 21) of PTH intervention (27.84 ± 4.39% vs 18.10 ± 4.18%, *p* < 0.01) (**Fig 5A**). The FE predicted tibial failure load was highly linearly correlated with the total tibial BMC for both the Wild (R^2^ = 0.89) and WildPTH (R^2^ = 0.91) groups (**Fig 5B**)

## Discussion

In the present study, the longitudinal effects of PTH intervention on the bone mineral content (BMC), bone tissue mineral density (TMD), bone volume (BV), tibial endosteal area (TEA), tibial periosteal area (TPA) and tibial failure load were investigated using *in vivo* μCT imaging, a novel spatiotemporal image analysis, and the finite element analysis technique.

Compared to previous preclinical animal studies [**9**–**13**], the novelties in the present study are the application of a novel spatiotemporal analysis approach and an analysis of the entire tibia instead of a small region of the tibia, such as the proximal region or the midshaft. The spatiotemporal analysis method provided more precise quantifications of bone changes [**15**], and the analysis in the entire tibia allows for the visualization of the progression of PTH intervention across the entire tibial region. For example, it is shown in the present study that the increase of TPA started from the midshaft region and then extended to most other regions after four weeks of PTH treatment. Compared to the authors’ previous studies [**14**–**15**], the present study advances the literature by providing information on the effects of PTH on the structural parameters of bone (BV, TEA and TPA) as well as information on the correlations between localized bone changes and the failure behaviors of bone.

In the present study, the BV, TMD and BMC data revealed the mechanism of how bone adapts its volume and mineralization after PTH intervention. It was revealed that after three weeks (week 20) of PTH intervention, the BV was significantly increased across the entire tibia, but the TMD maintained the same level throughout the intervention period, which implies that PTH is an anabolic drug mainly inducing changes of bone volume and that the bone mineralization cannot be altered by PTH intervention. The effect of PTH intervention on the BMC was similar to that on BV, possibly because in the present study, the BMC was calculated as the BV times their corresponding TMD values, which did not change throughout the intervention period. Another important finding is that after the PTH treatment, the increase of both the BV and BMC started in the proximal tibial region and then spread to the entire tibial region. One possible explanation is that there are many trabecular bones in the proximal tibial region which have a much more pronounced response to PTH treatment than do other regions, where mainly cortical bones are present. When comparing our results to the data from literature, our findings agree well [**29**–**31**]. Previous studies also found increased bone volume in PTH treated groups [**29–30**] and that the intraspecimen bone mineralization was not altered by PTH treatment in rats [**30**–**31**].

The data on the TEA and TPA revealed in detail the mechanism of how the tibia changes its geometry in the periosteal and endosteal areas after PTH intervention. It was revealed that the TEA decreased after two weeks of PTH intervention and the TPA increased after three weeks of intervention. These findings imply that the PTH intervention stimulated the formation of new bones in both the tibial endosteal and periosteal surfaces; consequently the tibial cortex thickness was increased. Previous studies also showed the increased tibial cortex thickness and strength after the application of PTH intervention [**29**–**31**]. However, the present study is the first to reveal how the bone cortex thickened in the endosteal and periosteal surfaces across the entire region. Additionally, it was found that after the PTH treatment, the increasing TPA started from the midshaft region, while there is no clear pattern for the increasing TEA. One possible explanation for the adaptation pattern of TPA is that the midshaft region of the mouse tibia bears more weight than other regions of the mouse tibia during every day activities [**32**], which stimulates more bone formation activities, in agreement with the bone mechano-regulation mechanism [**33**]. Regarding the effect of PTH on the TEA, it is still unclear which parameter is the main one that regulates the increase of TEA. Further investigations are needed.

The FE analysis revealed that the tibial failure loads significantly increased after three weeks of PTH intervention, while the increase of BV and BMC started locally just two weeks after PTH intervention, which implies that localized changes in the structural and densitometric parameters of bone are not sufficient to alter the global mechanical behaviors of the tibia, at least in the simulated compression scenario. The regression analysis revealed that the tibial failure loads are highly linearly correlated with the total tibial BMC, implying that the total tibial BMC is a crucial parameter for predicting changes in tibial failure loads. Because of the unique FE models (80% of the entire tibia) created in the present study, there is no literature available to validate the FE predictions. However, the trend predicted from the FE models, i.e., that PTH increased the bone strength, agrees well with the mechanical testing data reported in the literature [**34**–**35**]. Additionally, the correlation pattern between the mechanical properties of the tibia and the total BMC agrees well with previous studies, which also found that the total tibial BMC is highly linearly correlated with the tibial stiffness in both the PTH intervention and vehicle groups [**15**] and is highly linearly correlated with the tibial stiffness and failure load in both the ovariectomy and Sham mouse groups [**14**]. Therefore, in preclinical animal studies, the total tibial BMC may have the potential to replace the time consuming FE analysis to predict the mechanical properties of bone (stiffness and failure load).

Several limitations related to the present study should be noted. First, it is assumed that the tibial growth over one week can be ignored; thus a 3D rigid image registration method was used to register the image datasets obtained with a scanning interval of one week. Ideally, a full elastic registration method [**36**] should be used to remove the influence of skeletal growth on the results. However, on one hand, the application of a full elastic registration method is still under development. On the other hand, data from the literature showed that tibial growth of mice aged from 14 to 22 weeks old is stable [**37**–**38**] and that the rigid registration method performed in the present study in a stepwise manner could produce acceptable and relatively small errors [**33**]. Second, the tibia was chosen as the site of investigation in the present study because it is easier to set up a mouse tibia in the *in vivo* scanner. However, the femur has a higher fracture risk than the tibia in humans. Including the femur in the *in vivo* imaging would largely increase the scanning time, which would pose a large challenge for anesthetizing the mouse during the *in vivo* scanning. Additionally, the damage to the mouse bone induced by the imaging radiation would highly increase if the scanning time is increased. In the future, the same analysis approach should be applied separately to the mouse femur to see if the same mechanism can be found in the mouse femur and to provide potentially more clinically relevant values. Third, the influence of imaging radiation on the results is ignored in the present study. However, it is believed that the conclusions made in the present study will still be valid even through there are some inevitable radiation effects. This is because the authors’ previous study [**15**] showed that the radiation increased the BMC by 5% on average and produced similar effects for both PTH-treated and control mice. Additionally, the authors’ previous study showed that the intervention induced similar effects between the treated and control groups for the bone parameters investigated in the present study, i.e., TEA, TPA, BV, and TMD [**14**]. Furthermore, it should be noted that in the present study the PTH treatment was applied to the normal, not osteoporotic, female mice in order to isolate the effects of PTH on bone properties. Additionally, it should be noted that the application of a different dosage of PTH may alter the mechanism, but this effect is not investigated in the present study.

In summary, the present study revealed that after PTH treatment, the increases in the BMC and BV started locally in the proximal tibial region and then extended to the entire tibial region. However, the increase of TPA started locally in the midshaft region and then extended to other bone regions after four weeks of intervention. The localized changes in the structural and densitometric parameters of bone cannot lead to significant changes in tibial failure loads, and the tibial failure loads are highly linearly correlated with the total tibial BMC. The present study provided additional important insights into the mechanism of the effects of PTH intervention on whole bone behavior. Additionally, the spatiotemporal analysis approach can be used to conduct comprehensive investigations in bone research in other scenarios in the future.

## Ethical approval

All of the procedures of animal manipulation performed in the present study were approved by the Research Ethics Committee of Dalian University.
